# Dehydroepiandrosterone and Bone Health: Mechanisms and Insights

**DOI:** 10.3390/biomedicines12122780

**Published:** 2024-12-06

**Authors:** Nur-Vaizura Mohamad, Nur-Syahirah Che Razali, Nur-Amira Mohd Shamsuddin

**Affiliations:** 1 Centre for Drug and Herbal Development, Faculty of Pharmacy, Universiti Kebangsaan Malaysia, Jalan Raja Muda Abdul Aziz, Kuala Lumpur 50300, Malaysia; 2 Center for Toxicology and Health Risk Studies, Faculty of Health Sciences, Universiti Kebangsaan Malaysia, Jalan Raja Muda Abdul Aziz, Kuala Lumpur 50300, Malaysia; syahirahcherazali@gmail.com; 3 Centre for Drug Delivery Technology and Vaccine, Faculty of Pharmacy, Universiti Kebangsaan Malaysia, Jalan Raja Muda Abdul Aziz, Kuala Lumpur 50300, Malaysia; viumiera2@gmail.com

**Keywords:** dehydroepiandrosterone, sex steroid hormone, bone mineral density, adrenal glands

## Abstract

Background/Objectives: Dehydroepiandrosterone (DHEA), a steroid hormone produced by the adrenal glands, plays a key role in various physiological processes, including bone health. Its age-related decline is linked to reduced bone density, though the mechanisms by which DHEA affects bone metabolism remain complex. This review summarises the diverse effects of DHEA on bone metabolism and density, highlighting its therapeutic potential; Methods: A literature search on the effects of DHEA on bone-related parameters was conducted from PubMed and Scopus using a specific search string, and after removing duplicates and irrelevant articles, 36 relevant full-text studies were included; Results: DHEA promotes osteoblast differentiation and proliferation, regulates the RANKL/OPG ratio, and inhibits osteoclastogenesis and bone resorption. Its osteogenic effects are mediated through multiple signalling pathways. In ovariectomised rat models, DHEA enhances trabecular bone volume, stimulates osteoblast proliferation, and increases oestradiol production and aromatase activity. In elderly individuals with low androgen levels, DHEA supplementation increases sulphated DHEA and oestradiol levels and improves bone mineral density, particularly in the ultra-distal radius of women and the femoral neck of men. However, the clinical use of DHEA remains debated due to inconsistent study results. Its effects on bone health may vary based on factors such as age, gender, and health conditions, emphasising the need for further research to clarify its mechanisms and optimise its use; Conclusions: In conclusion, while DHEA shows potential as a modulator of bone health, comprehensive clinical trials are required to assess its efficacy and safety, particularly in at-risk populations.

## 1. Introduction

Dehydroepiandrosterone (DHEA) and its sulphate ester, DHEAS, are the most abundant circulating steroids found in humans. Both of these compounds exhibit pleiotropic effects on various aspects of human health, including ageing, bone health, metabolic diseases, neurological function, cancer, immune disorders, cardiovascular diseases, diabetes, muscle function, sexual dysfunction, and other health conditions [1,2]. DHEA is primarily synthesised from cholesterol in the adrenal glands, although smaller amounts are also produced by the testes, ovaries, skin, and brain. In contrast, DHEAS is exclusively derived from DHEA in the adrenal zona reticularis, a region characterised by high sulphuryl transferase activity [3]. The secretion of DHEA and DHEAS from adrenals is regulated by the hypothalamus–pituitary adrenal (HPA) axis and growth hormone–insulin-like growth factor-1(GH–IGF-1) axis [4]. DHEA can be transformed in peripheral tissues to more potent androgens and oestrogens by aromatase activity [5]. This local steroid production allows for the autoregulation of the hormonal environment according to specific local needs, minimising systemic effects that are known as intracrinology.

In premenopausal women, approximately 50–75% of oestrogens and the majority of androgens are generated through intracrine mechanisms utilising DHEA. Following menopause, particularly, all androgens and oestrogens are synthesised at the tissue level [6,7]. Bone health has garnered considerable attention, as numerous DHEA metabolites are recognised for their roles in bone homeostasis, particularly oestrogen and testosterone. Evidence from prior studies suggests that DHEA supplementation may enhance bone mineral density (BMD) and confer beneficial effects on bone health. BMD is a measurement that indicates the amount of minerals, mainly calcium, present in a specific volume of bone. It reflects the strength and density of bones. Higher BMD typically means stronger bones, while lower BMD can indicate weaker bones, increasing their susceptibility to fractures and conditions like osteoporosis. DHEA functions as a metabolic intermediate in the biosynthesis of sex hormones, with a past clouded in controversy and bold claims. It was once regarded as a miraculous substance, often called a fountain of youth, with the promise of curing a range of ailments. However, in the 1980s, the Food and Drug Administration (FDA) banned DHEA due to inadequate evidence supporting its health benefits and a lack of data on its long-term use. In 1994, DHEA was reintroduced to the market as a dietary supplement under the Dietary Supplement Health and Education Act, enabling consumers to access it without restrictions. The hormone was reintroduced in 1994 for open-market sale as a dietary supplement under the Dietary Supplement Health and Education Act. Since then, promising research has emerged, including randomised controlled trials and subsequent meta-analyses examining the potential benefits of DHEA across various conditions. Bone health continues to be a significant focus of this research, particularly given the established involvement of DHEA metabolites, especially oestrogen and testosterone, in maintaining bone homeostasis.

## 2. Literature Search

The literature was acquired using two electronic databases (PubMed and Scopus) in January 2024. The search string used for the literature search was “(Dehydroepiandrosterone OR “Dehydroepiandrosterone sulfate” OR “DHEA” OR DHEA-S) AND (bone OR osteoporosis OR fracture OR osteoblast OR osteoclast)”. All records from the inception of the databases were searched. Duplicates were removed and the titles of the remaining records were screened to remove reviews and irrelevant articles. From the remaining records, full-text articles were assessed for eligibility and a total of 36 articles were included based on the inclusion criteria. The current review includes all original research articles published between 2006 and 2021 with only articles published in English considered. The current review includes the effects of DHEA on bone-related parameters as primary outcomes across three experimental models. Firstly, human studies focus on adults with osteoporosis or postmenopausal women and examine the effects of DHEA supplementation on bone mineral density. Secondly, animal studies involving animal models of postmenopausal osteoporosis, such as ovariectomised rodents, assessment of outcomes such as bone mineral density, bone strength, or markers of bone turnover, and studies with controlled experimental designs. Finally, cell culture studies using relevant cell lines, such as osteoblasts, osteoclasts, or mesenchymal stem cells, assess outcomes like cell proliferation, differentiation, or the expression of bone-related markers.

## 3. Overview of DHEA

DHEA is a steroid hormone produced primarily by the adrenal glands, which are located on top of the kidneys. It is one of the most abundant steroid hormones in the human body and serves as a precursor for the synthesis of other hormones. The testes or ovaries also produce these hormones to a limited extent and may be converted to androgens (male sex hormones) and oestrogens (female sex hormones) in peripheral tissues. DHEA has an additional sulphate molecule attached to it, which is the primary distinction between it, known as DHEAS. The human body naturally makes DHEA and can convert it into a range of hormones, including androgens and oestrogens. DHEA is also available in supplements, made synthetically using chemicals found in soy and wild yam. In the United States, DHEA is sold as an over-the-counter supplement and medication called prasterone.

### 3.1. DHEA Synthesis and Metabolism

DHEA is potentiated locally via conversion into testosterone and dihydrotestosterone (DHT) in the skin and hair follicles [8]. DHEA can be converted into weak oestrogen and transformed into potent oestrogens such as oestradiol in certain tissues like the vagina and thereby producing oestrogenic effects in such tissues [8]. DHEA is synthesised from cholesterol via the enzyme’s cholesterol side-chain cleavage enzyme (CYP11A1: P450scc) and 17α hydroxylase/17,20-lyase (CYP17A1), with pregnenolone and 17α-hydroxypregnenolone as intermediates. It is derived mostly from the adrenal cortex, with only about 10% secreted from the gonads. DHEA is produced by the adrenal glands in humans and is taken up by several tissues, including the brain, liver, kidney, and gonads, where it is metabolised to 5-androstene-3β,17β-diol, 4-androstene-3,17-dione, testosterone, oestrogen, and other biologically active steroids, depending on the tissue [9]. DHEA is transformed into DHEAS by sulphation at the C3β position via the sulfotransferase enzymes sulfotransferase 2A1 (SULT2A1) and, to a lesser extent, sulfotransferase 1E1 (SULT1E1). DHEAS is almost exclusively produced in the adrenal glands, whereas DHEA, androstenedione, and testosterone are produced in the adrenal glands, ovaries, and by peripheral conversion.

DHEA is primarily produced by the adrenal glands, although it can also be synthesised in small amounts by the gonads, which include the testes and ovaries. This hormone is subsequently converted to androstenedione by the enzyme 3β-hydroxysteroid dehydrogenase (3β-HSD). Following this conversion, androstenedione can be transformed into testosterone through the action of the enzyme 17β-hydroxysteroid dehydrogenase (17β-HSD). Additionally, testosterone can be converted back to androstenedione via a reverse reaction facilitated by 17β-HSD. Furthermore, the enzyme 5α-reductase is capable of converting testosterone into the more potent androgen known as DHT. In summary, DHEA functions as a precursor that can be metabolised into testosterone through a series of enzymatic conversions, primarily involving the enzymes 3β-HSD and 17β-HSD, thereby allowing DHEA to contribute to the production of testosterone and other androgens in the body. In target tissues, circulating testosterone is converted to a more potent androgen, DHT, by 5α-reductase and aromatised to oestradiol. DHT exhibits a higher affinity for androgen receptors (ARs), resulting in more pronounced androgenic effects compared to testosterone. This conversion is a vital mechanism for amplifying androgen signalling in specific tissues. In addition, testosterone can also be converted to oestradiol (E2) through the action of the enzyme aromatase (CYP19A1). Aromatase is expressed in various tissues, including the gonads, adipose tissue, and brain [10]. The process of aromatisation, where testosterone is transformed into oestradiol, serves as an essential pathway for converting androgens to oestrogens. This conversion plays a crucial role in various physiological processes, such as reproductive function and bone health.

Several recent studies have shown that type II 3beta-hydroxysteroid dehydrogenase (HSD3B2), cytochrome b5 (CYB5), and SULT2A1 play an important role in the regulation of adrenal androgen production [11,12]. HSD3B2 is the key enzyme that converts pregnenolone and 17α-hydroxypregnenolone into progesterone and 17α-hydroxyprogesterone, respectively. This conversion is vital for the biosynthesis of adrenal androgens, guiding steroid precursors toward the androgen production pathway. To enhance this process, CYB5 acts as a cofactor, boosting the activity of the enzyme CYP17A1. CYP17A1 facilitates the conversion of 17α-hydroxypregnenolone and 17α-hydroxyprogesterone into DHEA and androstenedione. The presence of CYB5 increases the 17,20-lyase activity of CYP17A1, promoting the production of adrenal androgens. Additionally, SULT2A1 sulphonates DHEA, converting it into DHEAS, the primary circulating form of DHEA. DHEAS serves as a reservoir for the production of more potent androgens. In postmenopausal women, the production of oestrogens and DHEA from the ovaries is near zero [6], making adrenals the main source of oestrogens and testosterone through DHEA. It has been reported that androstenediol levels increase from early perimenopause to late perimenopause [13]. Androstenediol is a weak androgen that can be converted into more potent androgens, such as testosterone, by the enzyme 17β-HSD, or into oestrogens by CYP19A1. A study by Lasley et al. found that the levels of circulating androstenediol are insufficient to act as an oestrogen in premenopausal women. However, in early perimenopause, the concentration of androstenediol, which rises due to increased DHEAS levels, surpasses that of oestradiol [14].

**Figure 1:**
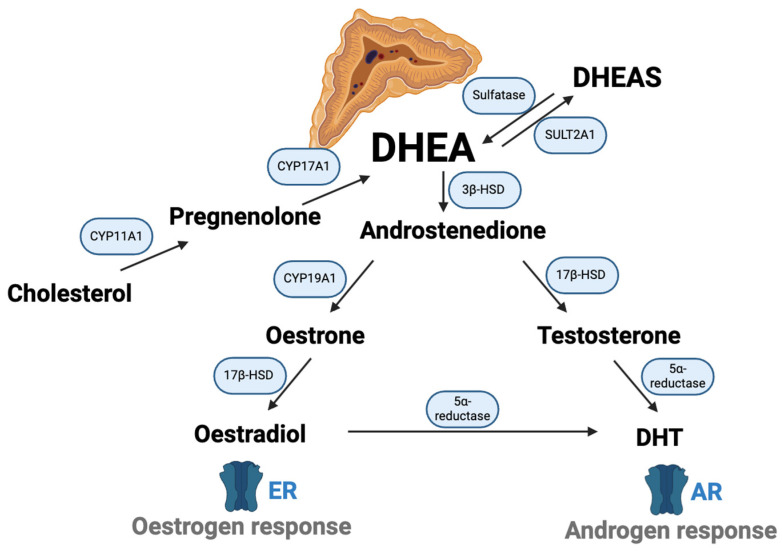
DHEA metabolic cascade: enzymatic transformations to steroid hormones. Abbreviation: AR, androgen receptor; CYP11A1, cholesterol side-chain cleavage enzyme; CYP17A1, 17α-hydroxylase/17,20-lyase; CYP19A1, aromatase; DHEA, dehydroepiandrosterone; DHEAS, DHEA sulphated; ER, oestrogen receptor; SULT2A1, sulfotransferase 2A1; 3β-hydroxysteroid dehydrogenase; 17β-HSD, 7β-hydroxysteroid dehydrogenase; 3β-HSD. Created in BioRender. Chua, E. (2024) https://BioRender.com/q31o388 (accessed on 23 October 2024).

The synthesis of DHEA is regulated by various factors, including adrenocorticotropic hormone (ACTH) secreted by the pituitary gland. The secretion of ACTH, in turn, is regulated by the HPA axis, which responds to stress and circadian rhythms. DHEA can be further metabolised in various tissues and organs to produce biologically active hormones. Some of the key metabolites include androstenedione, which is an intermediate that can be converted into testosterone or oestrone (oestrogen) in peripheral tissues [3]. Figure 1 illustrates the biochemical pathways involved in the conversion of cholesterol to various steroid hormones, with a focus on DHEA and its derivatives. DHEA is converted into oestradiol by the enzyme 17β-HSD, while testosterone is transformed into DHT by 5α-reductase. Oestradiol binds to oestrogen receptors (ERs) to induce oestrogenic effects, whereas DHT interacts with AR to produce androgenic effects.

DHEA has various physiological roles in the body, including supporting overall well-being and vitality, modulating the immune system, promoting bone health, and affecting mood and cognitive function. It also plays a role in the development of secondary sexual characteristics during puberty. Studies indicate that DHEAS is associated with increased BMD and decreased fracture risk, suggesting that it plays a protective role by enhancing bone mass [15]. Additionally, a study by Ouanes et al. found that dysregulation of the HPA axis, marked by high cortisol and low DHEAS levels, is linked to Alzheimer’s disease pathology, including tau pathology, neurodegeneration, and clinical symptom progression, underscoring DHEAS’s role in cognitive function [16]. DHEA levels in the body tend to peak in the late teens to early twenties and gradually decline with age. By the time people reach their 70s and 80s, DHEA levels are significantly lower than in their youth. DHEA supplements are available and are sometimes used for various purposes, including hormone replacement therapy, anti-ageing, and mood regulation. However, the use of DHEA supplements should be approached with caution and under medical supervision, as excessive intake can have adverse effects.

In summary, DHEA is a steroid hormone synthesised from cholesterol in the adrenal glands. It serves as a precursor for other hormones and plays a role in various physiological functions. Its synthesis and metabolism are tightly regulated, and age-related changes in DHEA levels can impact health and well-being.

### 3.2. DHEA on Androgen and Oestrogen Receptors

DHEA exerts many of its effects via the sex steroid receptors AR or ER after peripheral enzymatic conversion to androgens or oestrogens within target cells [17,18]. There are two ER subtypes (ERα and ERβ) that are differentially expressed in different tissues, whereas ARs are ubiquitously expressed in all tissues. The AR and ERs function mainly as nuclear receptors and can affect gene transcription in target cells.

DHEA activated transcription of the ERβ in a more physiologically relevant range of 500 nM in contrast to the micromolar concentrations required to antagonise the AR. Chen et al. observed that DHEA exerted clear-cut agonistic effects on ERβ. DHEA strongly activated transcriptional activity in a construct containing ERβ, which was 50% higher than that elicited by oestradiol (E2) [19]. Moreover, DHEA administration elicited greater transcriptional activation of ERβ vs. ERα and no detectable activation of AR, suggesting that ERβ is the preferred target for the transcriptional effects of DHEA. Treatment of granulosa cells with DHEAS resulted in the upregulation of enzymes and receptors involved in androgen and oestrogen metabolism, including the AR, CYP19A1 and 3β-HSD, while simultaneously downregulating ERβ. This treatment also led to increased production of the oestrogens, oestrone and oestradiol, with no significant changes observed in the levels of progesterone, androstenedione, or testosterone. These findings suggest that DHEAS can modulate the expression of key steroidogenic enzymes and receptors in granulosa cells, enhancing oestrogen production while having minimal effects on other steroid hormones [20]. Studies demonstrated that the effect of DHEA on trabecular BMD and thymic atrophy does not require signalling via the AR and that DHEA can activate the classical oestrogen signalling pathway in bone and thymus in sex steroid-deficient or male mice [17]. These findings indicate that ERs, and not the AR, are important for the action on bone and thymus by DHEA under the present experimental conditions. In contrast, the induction of an androgenic phenotype in the submandibular glands by DHEA is dependent on the AR. Thus, both ERs and the AR are important for mediating the effects of DHEA in an organ-dependent manner. However, the authors cannot exclude the possibility that DHEA may act on an as-yet unknown steroid receptor or via the suggested membrane-bound nonnuclear DHEA receptor. DHEA metabolites have been found to directly interact with oestrogen receptors (ERα and ERβ) in several ways. DHEA metabolites have been observed to compete with 17β-oestradiol for binding to both ERα and ERβ, suggesting that they can directly bind to and potentially modulate the activity of these ER subtypes. Additionally, DHEA metabolites have been shown to stimulate the proliferation of MCF-7 breast cancer cells, which are known to be responsive to oestrogen signalling [21]. This indicated that DHEA metabolites can elicit oestrogenic-like effects on target cells, likely through their direct interaction with the ER. Furthermore, the direct binding of DHEA metabolites to the ER has been found to modulate the expression of oestrogen target genes in vivo, suggesting that DHEA metabolites can directly impact the transcriptional activity of the ER and the downstream regulation of oestrogen-responsive genes. These findings showed that DHEA metabolites can interact directly with both ERα and ERβ, competing for binding with the endogenous oestrogen, 17β-oestradiol. This direct interaction with the ER allows DHEA metabolites to exert oestrogenic-like effects.

## 4. Effects of DHEA on Bone

DHEA has been shown to positively impact bone health through various mechanisms. Cell-based (in vitro) and animal (in vivo) studies have demonstrated DHEA’s ability to promote osteoblast activity and bone formation, while human clinical trials have reported associations between DHEA levels and improved BMD and reduced fracture risk.

### 4.1. Effects of DHEA in In Vitro (Cell-Based) Studies

The effects of DHEA have been previously investigated in bone cells and are summarised in Table 1. In an earlier study by Harding et al., the impact of DHEA on osteoclastogenesis pathways was explored using a human clonal osteoblastic cell line (HCC1) [22]. Recent findings indicate that treatment with DHEA significantly influences the expression of osteoprotegerin (OPG), a key regulator in bone metabolism. OPG serves as a decoy receptor for nuclear factor kappa-Β ligand (RANKL), which plays a crucial role in regulating osteoclastic development and activity; thus, the RANKL/OPG ratio is considered the primary determinant of bone resorption. The results also demonstrated that DHEA treatment in HCC1 downregulated several inflammatory cytokines, including interleukin (IL)-4, IL-6, and interferon-cc (IFN-cc). Meanwhile, genes such as Notch 2, the insulin receptor, and thrombin receptor (PAR1), which are potential stimulators of osteoblast proliferation and differentiation, were found to be upregulated. Notch 2 is thought to be a positive regulator of osteoblastic differentiation in multipotent mesenchymal cells, while insulin receptors on bone cells modulate the synthesis of collagen. PAR-1, present on osteoblasts, is therefore involved in mediating the proliferative effects of thrombin. Additionally, DHEA significantly promoted the proliferation of osteoblasts while inhibiting their apoptosis in murine osteoblastic cells via a mitogen-activated protein kinase (MAPK) signalling pathway that is independent of both the AR and ERs [23]. Similarly, a study by Malik et al. reported that treatment with 5-androstene-3β,7β,17β-triol (β-AET), an active metabolite of DHEA, significantly reversed glucocorticoid (GC)-induced suppression of IL-6, IL-8, and OPG expression by human osteoblast-like MG63 cells, and induced osteoblast differentiation in human bone marrow-derived mesenchymal stem cells (hBM-MSCs) [24]. In a mouse thermal injury model, β-AET treatment mitigated the loss of bone mineralisation, restored chondrocyte-mediated endochondral bone growth, and slowed osteoclast-mediated cortical bone erosion. DHEA was also reported to be able to modulate the cell viability of osteoblasts in the study by Wang et al. through the dependence on the presence or absence of ERβ [25]. When the level of ERβ was high, DHEA significantly amplified the proliferation of hMG63 osteoblast cells. However, when the expression of ERβ was silenced, DHEA had no significant effect on the viability of hMG63 cells. Production of OPG was also significantly elevated by DHEA treatment, while the levels of RANKL and ERα were unaffected.

A study by Kaivisoja et al. reported that DHEA decreased the viability rate of hBM-MSCs and osteoblastic SaOS-2 cells in a concentration-dependent manner [26]. Treatment with DHEA, following hBM-MSC-mediated and 5α-reductase-dependent conversion to dihydrotestosterone, clearly promoted the osteoinduction of MSCs induced with β-glycerophosphate, ascorbate, and dexamethasone. The levels of alkaline phosphatase (ALP), suppressor of mothers against decapentaplegic 1 (SMAD1), runt-related transcription factor 2 (RUNX2), osteopontin (OP), and osteocalcin (OC) RNA were found to be increased. Furthermore, DHEA raised the mRNA levels of OP and OC in non-induced hBM-MSCs. Another study that explored the effects of DHEA in osteoblast differentiation using MSCs demonstrated an increase in osteoblast production and a significant increase in the ALP activity of osteoblasts in MSCs cultured in osteoblastogenic medium after treatment with DHEA [27]. The expression of RUNX2 and osterix (OSX), which are the transcription factors associated with osteoblastogenesis, were also significantly elevated after DHEA treatment, thereby increasing the expression of OC and collagen 1 (COL1) genes that are expressed in osteoblasts. DHEA increases the number of Foxp3^+^ Tregs, thus increasing the osteoblast differentiation.

In both primary cultures and a cell line of hBM-MSCs, DHEA stimulated osteoblastogenesis, evidenced by significant inductions of ALP activity and osteoblast gene [28]. This stimulation of insulin-like growth factor-1 (IGF-1) gene expression and osteoblastogenesis in hBM-MSCs requires the IGF-1 receptor, phosphoinositide 3-kinase (PI3K), mitogen-activated protein kinase (p38 MAPK), or p42/44 MAPK signalling pathways as confirmed through the induction of osteoblast marker genes, ALP, RUNX2, and COL1 after blocking the IGF-1 activity. This indicated that the IGF-1 signalling pathway is a crucial mediator of DHEA’s bone-forming effects. However, DHEA likely operates through multiple interconnected pathways to promote osteoblast differentiation and bone mineralisation. Other findings illustrated that DHEA treatment in hBM-MSCs significantly induced the expression of mRNA of bone morphogenetic protein 2 (BMP2), RUNX2, and scalable processor architecture (SPARC), which are the main genes that are involved in osteogenic differentiation [29]. The upregulation of these key osteogenic genes provides mechanistic insights into how DHEA could help restore bone health by promoting osteoblast differentiation from mesenchymal stem cell precursors. Similarly, the latest study investigating the role of DHEA in facilitating osteoblast differentiation was then conducted, whereby the DHEA mechanism was explored in hBM-MSCs together with osteogenic induction medium (OIM) [30]. The results showed that OIM with DHEA could significantly promote proliferation and osteogenic differentiation with a significant increase in ALP activity, BMP2, RUNX2, and COL1a1 expressions of hBM-MSCs than OIM alone suggests DHEA may be a promising therapeutic target for improving bone formation and regeneration. The increase in osteoblast proliferation and differentiation induced by DHEA treatment was also observed in a study using calvarial osteoblasts isolated from female rats [31]. Likewise, the results showed that DHEA treatment led to a significant increase in osteoblast proliferation and ALP activity, along with elevated expressions of ALPL and OSX. These findings on the differentiation-related genes further confirm that DHEA can promote osteoblast differentiation. The observation that these effects occurred in calvarial osteoblasts rather than mesenchymal stem cells suggests that DHEA’s bone-forming actions extend beyond the stem cell stage and can directly affect mature osteoblasts.

On the contrary, a study by Giu et al. reported that the treatment of DHEA did not significantly affect the cell viability of bone marrow-derived monocyte/macrophage precursor cells (BMMs) from the femurs and tibias of 20-week-old mice in the ovariectomised (OVX) [32]. Nevertheless, DHEA inhibited the osteoclastogenesis of BMMs through significant inhibition of TRAP-positive cell formation, suggesting that DHEA may have a suppressive effect on osteoclast differentiation and function. Additionally, a study by Sun et al. reported that DHEA suppressed bone growth by acting directly at the growth plate through ERs in metatarsal bone rudiments isolated from Sprague Dawley rat foetuses [33]. This growth inhibition is mediated by decreased chondrocyte proliferation and hypertrophy/differentiation, along with increased chondrocyte apoptosis, primarily through the ER via the nuclear factor kappa B (NF-κB) signalling pathway.

Infection with *Brucella abortus* (*B. abortus*) induced apoptosis and inhibited osteoblast function in the mouse MC3T3-E1 osteoblast cell line. The role of DHEA in promoting the proliferation of osteoblast was further clarified in a study by Gentilini et al. with the infection of *B. abortus* in mouse clonal MC3T3-E1 cells [34]. DHEA treatment was able to significantly reverse these negative effects of *B. abortus* infection on osteoblasts by reducing the levels of the glucocorticoid receptor (GRα/β) and the 11β-hydroxysteroid dehydrogenase type 1 and 2 (11β-HSD1/2) ratio in the infected osteoblasts, suggesting the protective role of DHEA in preventing bone loss. However, a study by Pesce Viglietti et al. demonstrated that DHEA did not have a significant effect on osteoclast differentiation in *B. abortus*-infected osteocytes of the MLO-Y4 cell line [35]. DHEA also did not significantly affect the levels of inflammatory markers like tumour necrosis factor- α (TNF-α), IL-6, RANKL, and matrix metalloproteinase-2 (MMP-2) in the infected osteocytes. These contrasting findings highlight the cell-type-specific effects of DHEA on bone cells and the need for further research to fully elucidate its mechanisms of action.

### 4.2. Effects of DHEA in In Vivo (Animal) Studies

The effects of DHEA on bone-related parameters in animal studies has been summarised in Table 2. A study utilising ovariectomised (OVX) inbred strains of BALB/c mice to replicate the impact of reduced ovarian function on bone health found that the administration of 5 mg/kg per day of DHEA, either orally or intragastrically for 90 days, resulted in a transformation of bone tissue morphometry. This included an increase in trabecular bone volume (BV) in the vertebrae and femur of OVX mice. DHEA also promoted the proliferation of osteoblasts (OBs), as indicated by increased fluorescence intensity and enhanced expression of proliferating cell nuclear antigen (PCNA) in OBs, as demonstrated through flow cytometric analysis [23]. Furthermore, DHEA treatment significantly elevated oestradiol production by tibia fragments and aromatase activity, thereby contributing to an increased oestrogen concentration within the bone [36]. It was postulated that a significantly higher serum concentration of DHEA and E2 was found in OVX female Sprague Dawley rats that were given 20 mg/kg body weight of DHEA intraperitoneally for 8 weeks [37,38,39]. The findings also indicated that DHEA elevated the BMD of the tibial proximal metaphysis (cancellous site) and local DHT [37,39]. However, there were no significant differences in the BMD of the tibial diaphysis (cortical-abundant region) and free testosterone concentration in the tibial proximal metaphysis observed. A significant increase in the bone mineral contents (BMCs) of the lumbar spine (trabecular-abundant region) and BMD of the lumbar spine and femoral wet weight were shown in OVX rats with DHEA treatment, while no difference in the long and short width and length of the femur and breaking force of the femoral diaphysis site (cortical bone-abundant) were observed [38]. These findings suggest that DHEA treatment in OVX rats had a more pronounced effect on the trabecular bone-rich lumbar spine, as evidenced by the increases in BMC and BMD, compared to the cortical bone-dominant femoral diaphysis, which did not show significant changes in morphology or strength.

Oral administration of 5 mg/kg per day of DHEA for 3 months in OVX C57BL/6 mice has improved bone morphology in mice with osteoporosis by increasing the BV, BMD, and trabecular number (Tb.N) in the bone while decreasing the trabecular spacing (Tb.Sp) compared to OVX treated with saline [27]. Similar treatments were given intragastrically on OVXBALB/c mice as the results showed that DHEA significantly increased the BMD in the femur and vertebra [40]. The treatment enhanced osteoblast differentiation by increasing the bone mass and formation (bone volume/tissue volume (BV/TV), osteoblast surface/bone surface (Ob.S/BS), mineralising surface/bone surface (MS/BS), and bone formation rate) through analysis of the dynamic and static bone histomorphometric parameters. It also significantly decreased the osteoclast-related parameters, which include osteoid surface/bone surface (OS/BS), osteoclast number/bone perimeter (Oc.N/BP), and eroded surface/bone surface (ES/BS). This result proved that DHEA alleviated OVX-induced bone loss by inhibiting osteoclast-mediated bone resorption and by improving osteoblast-mediated bone formation. DHEA treatment was also found to influence the endocrine–immune network in postmenopausal osteoporosis, specifically by affecting CD4+ T cells. DHEA led to a significant reduction in the OVX-induced expansion of CD4+ T cells in both the bone marrow and spleen, as well as a significant decrease in the production of the pro-inflammatory cytokine TNF-α. Additionally, DHEA mitigated the OVX-induced increase in CD4+ T cell populations and suppressed TNF-α production, a key inflammatory cytokine known to promote osteoclastogenesis and bone resorption.

A study by Malik et al. that used β-AET, an active metabolite of DHEA, as a treatment for thermal trauma-induced bone loss in male BALB/c mice has been proven to preserve BMC [24]. Treatment with 50 mg/kg of β-AET maintained the BMC by opposing the thermal trauma-induced attenuation of normal bone growth and mineralisation, as indicated by the wet, dry, and ash weights of the femur bone. Treatment insignificantly increased the percent bone with a strong trend, as well as lowered the perimeter/area ratio. The effects of β-AET culminated in the preservation of the endochondral growth rate, as measured at the tibial epiphyseal growth plate, which was significantly higher in the 50 mg/kg treatment group. The β-AET also prevented bone erosion at the mid-diaphyseal region of the femur in thermal trauma-induced bone loss mice. The restored whole-body bone mineral content and endochondral growth suggest a reversal of glucocorticoid-mediated decreases in chondrocyte proliferation, maturation, and osteogenesis in the growth plate. The active DHEA metabolite, β-AET, has the potential to mitigate the detrimental effects of thermal trauma on bone, preserving the bone mineral content, microstructure, and endochondral growth, highlighting the therapeutic promise of DHEA and its metabolites in the context of trauma-induced bone loss. In another model of prednisolone-induced osteoporotic rats, treatment with DHEA at 250 mg/kg body weight orally, three times per week for 6 months showed a significant effect on bone biomarkers and microstructure [41]. DHEA treatment increased the serum levels of OC, 1,25-dihydroxyvitamin D3, and OPG while it decreased the serum levels of parathyroid hormone (PTH) and RANKL. DHEA acts as a protective measure resulting in the restoration of intact epiphyseal bony structure and articular surface. DHEA regulation on glucocorticoid activity and androgenic action provided a potent effect on bone. The effect of DHEA hormone as protection or for the treatment of osteoporosis revealed a significant improvement in OC and 1,25-dihydroxyvitamin D3 levels compared to the osteoporotic without treatment. As a protective measure, DHEA significantly lowers the PTH level but is insignificant as a therapeutic in osteoporotic rats. DHEA caused a significant increase in OPG associated with a significant decrease in RANKL levels. Rats that were orally administered 250 mg/kg body weight of DHEA for 6 months exhibited normal trabecular bone and epiphyseal bony structures in the left femur. Rats receiving DHEA for protective purposes showed intact epiphyseal bony structures and articular surfaces. However, when DHEA was administered as a treatment, it resulted in some necrosis and atrophy in the epiphysis.

The subcutaneously implanted DHEA at 50 mg for 12 weeks on orchidectomised male Wistar-Kyoto rats significantly increased the plasma testosterone level and PTH level compared to the orchidectomised rats without treatment [42]. This treatment has significantly increased the BMD of the tibia and BMC. Regarding bone remodelling markers, DHEA significantly increased the level of bone formation markers, including OC and ALP. Furthermore, treatment significantly increased the serum level of bone resorption markers, such as deoxypyridinoline (DPD) and tartrate-resistant acid phosphatase 5b (TRAP-5b). DHEA effects on the OPG/RANKL axis are shown by an increase in OPG while suppressing the RANK level. In terms of bone microarchitecture, DHEA reversed the reduction and thinning of trabeculae and empty bone lacunae by showing normal trabecular bone architecture with osteoblasts at the endosteal surface. These findings indicated that DHEA treatment in orchidectomised rats can restore hormonal balance by increasing testosterone and PTH levels, improve bone mineral density and content, enhance bone remodelling by boosting markers for both formation and resorption, regulate the OPG/RANKL axis to maintain bone health, and preserve normal trabecular bone structure.

### 4.3. Effects of DHEA on Human Studies

Previous evidence has shown the association of DHEA with bone health in human studies (Table 3). A randomised, placebo-controlled, double-blind study was conducted over 24 months to assess the effects of DHEA replacement on BMD in elderly individuals with low androgen levels [43]. The study involved 87 men and 57 women, averaging 60 years of age, who had low levels of DHEA. Participants were given a daily dose of 75 mg of DHEA for men and 50 mg for women. The findings revealed that DHEA supplementation significantly increased sulphated DHEA and oestradiol levels in both total and bioavailable forms. Additionally, DHEA replacement significantly increased BMD in the ultra-distal radius in women and the femoral neck in men. This indicates that DHEA replacement was able to restore the depleted DHEA and oestradiol levels as well as help maintain and potentially improve bone density in elderly individuals with age-related decline in sex hormone levels. In another randomised, double-blind study involving 70 women and 70 men aged 60–88 years with low serum DHEAS levels, participants were administered 50 mg of oral DHEA daily for one year to assess its impact on BMD [44]. The results indicated that DHEA significantly improved BMD in the total hip, trochanter, and shaft subregions compared to the placebo group. Notably, women experienced a significantly greater increase in lumbar spine BMD than men. Secondary compliance analyses also revealed a significant increase in BMD in the hip regions for those in the DHEA group. This finding is consistent with the study by Bácsi et al., which reported a positive correlation between serum DHEAS levels and BMD at both the lumbar spine and femoral neck in a cross-sectional study of 319 postmenopausal women [45].

A long-term study on DHEA replacement for primary adrenal insufficiency was conducted using a double-blind, randomised trial involving 106 subjects of both genders with an average age of 46 years who had Addison’s disease [46]. In this study, participants also received oral micronised DHEA at a dose of 50 mg daily for 12 months. A significant reversal of the progression of femoral neck BMD reduction was observed in those receiving DHEA. Gender-based analyses revealed a differential effect at the proximal radius, with a significant enhancement in BMD in men but not in women. Another similar study with low serum DHEAS levels was conducted to determine the effects of DHEA therapy on changes in sex hormones and IGF-1 and their associations with changes in BMD [46]. Subjects were randomly assigned with stratification by sex to receive oral DHEA 50 mg/day or placebo for one year. In comparison with the placebo group, participants receiving DHEA experienced a significant increase in hip and lumbar spine BMD. DHEA replacement increased the serum levels of DHEAS, testosterone, free testosterone index, oestrone (E1), E2, free oestrogen index (FEI), and IGF-1, while decreasing sex hormone-binding globulin (SHBG) in women. In men, DHEA treatment increased DHEAS, E1, FEI, and E2 and decreased SHBG. Additionally, DHEA treatment reduced the serum levels of C-terminal telopeptide of type 1 collagen (CTX) and significantly decreased bone-specific ALP levels compared to the placebo group. The study found that DHEA treatment increased BMD at the hip and lumbar spine in older adults with low DHEAS levels. It also affects sex hormone and IGF-1 levels differently in men and women while reducing markers of bone resorption and formation, suggesting a positive impact on bone turnover. A prospective, randomised, double-blind, placebo-controlled crossover trial conducted by Marder et al. examined the effects of DHEA on markers of bone turnover in premenopausal women with systemic lupus erythematosus (SLE) [47]. Thirteen premenopausal female patients with an SLE disease activity index (SLEDAI) score of less than 8 and an average age of 38.7 years participated in a crossover trial involving 22 weeks of treatment with prasterone (DHEA, 200 mg/day). DHEA supplementation increased the levels of RANKL, a key mediator of osteoclast-driven bone resorption, in premenopausal women with SLE. However, the treatment did not significantly affect the RANKL/OPG ratio or OC, a marker of bone formation. These findings suggest that DHEA may differentially impact specific markers of bone turnover in premenopausal women with SLE, with a more pronounced effect on pathways related to bone resorption. An increase in DHEAS levels is associated with less bone loss at the femoral neck and lumbar spine, as demonstrated by Ghebre et al. in a prospective population-based longitudinal study involving 1003 postmenopausal women with an average age of 54.7 years [48]. DHEA replacement therapy plays a beneficial role in treating steroid-induced osteoporosis in postmenopausal women. In a cross-sectional study conducted by Papierska et al., 19 women aged 50 to 78 years who had been on long-term glucocorticoid treatment for at least 2 years after menopause were examined. The results showed that taking oral micronised DHEA at doses of 25 to 50 mg daily led to significant increases in the serum levels of DHEAS, androstenedione, and testosterone after both 6 weeks and 6 months of treatment [49]. Additionally, during DHEA treatment, the serum concentrations of IGF-1 and OC also increased. After the duration of DHEA replacement therapy, BMD showed an increment in both the lumbar spine and femoral neck compared to the calcium and vitamin D phases, suggesting that DHEA replacement may play a beneficial role in the treatment of steroid-induced osteoporosis in postmenopausal women, potentially by modulating hormonal and growth factor pathways involved in bone metabolism.

In a different study, researchers analysed the relationship between the serum DHEAS levels and bone mass in men, which involved 1300 consecutive Korean men with an average age of 54.1 years [50]. The findings revealed a positive correlation between the serum DHEAS levels and BMD across all skeletal sites. Notably, the BMD values at the lumbar spine, total femur, femoral neck, and trochanter significantly increased in a dose-dependent manner as the DHEAS quartile categories rose from the lowest (Q1) to the highest (Q4). A study by Park et al. also demonstrated a positive correlation between the serum DHEAS level and IGF-1 level with BMD at various sites [51]. It is found that the BMD at the femur neck, at the ward, and the trochanter increased in a dose-dependent manner across increasing DHEAS tertile categories. In comparison with subjects in the lowest DHEAS tertile category (T1), those in the higher DHEA-S categories (T2, T3) showed significantly higher BMD at the femur neck, at the ward, and the trochanter. An international multicentre, prospective study, which included older men in three countries (United States, Sweden, and Hong Kong) with an average age of 75.5 years, proved that serum DHEAS marginally associated with BMD in the femoral neck and lumbar spine [52]. A cross-sectional analysis from a prospective multicentre study in Korea, which included 77 premenopausal women (average age 38.6–42.5 years), 237 postmenopausal women (average age 59.4–59.7 years), and 481 men (average age 55.3–56.9 years), was conducted [53]. The results revealed that the DHEAS levels were positively associated with BMD at the lumbar spine in both postmenopausal women and men. In contrast, the cortisol to DHEAS ratio was inversely associated with lumbar spine BMD in both groups. This suggests that maintaining an appropriate balance between cortisol and DHEAS may be important for preserving bone health, particularly in older adults. The lack of significant associations in premenopausal women indicates that the relationships between adrenal hormones and bone may be more pronounced in the postmenopausal state. In a study by Jankowski et al., data from four double-blind, randomised controlled trials with low DHEAS levels were analysed [54]. The subjects took 50 mg of oral DHEA daily for 12 months and the results showed that the treatment increased the serum DHEAS levels in both women and men. Women experienced increases in serum testosterone, E2, and IGF-1, along with a decrease in SHBG. In men, DHEA treatment led to increased serum E2 and IGF-1 and a decrease in SHBG. While DHEA therapy increased or slowed the decline in the lumbar spine, total hip, and trochanter BMD compared to the placebo group, there was no significant difference in BMD between DHEA-treated and placebo-treated men. DHEA supplementation had a positive impact on preserving or attenuating the decline in BMD at the lumbar spine, total hip, and trochanter in women but not in men. The bone tissue in the maxillary sinus is particularly sensitive to changes in DHEAS levels. This finding is supported by a cross-sectional study involving 63 perimenopausal women aged 45 to 55 years, all of whom had experienced amenorrhea for less than 2 years [55]. The study reported a strong positive correlation (r = 0.73) between the minimal bone density of the maxillary sinus and DHEAS levels. In another cross-sectional study of healthy postmenopausal women, higher DHEA levels were linked to improved physical function, indicated by stronger handgrip strength and faster gait speed [56]. Additionally, the DHEA levels positively correlated with BMD at the lumbar spine and total hip. These findings suggest that maintaining adequate DHEA levels may be crucial for preserving physical function and bone health in postmenopausal women. In premenopausal women, the ovaries produce sufficient oestrogen, which plays a critical role in maintaining bone density by inhibiting osteoclastogenesis. DHEA may act as a precursor to oestradiol, potentially enhancing its levels and supporting bone health in this population. In contrast, postmenopausal women experience a significant decline in oestrogen levels, leading to increased bone resorption and a higher risk of osteoporosis. In this context, the conversion of DHEA to oestradiol becomes crucial for compensating for oestrogen loss, potentially mitigating bone loss. DHEA stimulates aromatase activity, increasing the conversion of androgens to oestrogens. The modest rise in oestradiol levels through DHEA supplementation may benefit bone metabolism and help preserve bone density. In men with low androgen levels, DHEA can serve as a source of both androgens and oestrogens, potentially improving bone metabolism. While men primarily rely on testosterone for bone health, the conversion of DHEA to oestradiol also contributes to maintaining bone density, especially as testosterone levels decline with age. The complexities of bone metabolism and the interplay among various hormones highlight that, while DHEA can influence oestradiol levels, individual responses may vary based on factors such as baseline hormone levels, age, and overall health. Consequently, the impact of DHEA on bone metabolism may not be uniform across all individuals.

Currently, there are few long-term randomised controlled trials specifically evaluating the effects of DHEA supplementation on BMD and fracture prevention. Most studies focus on short- to medium-term outcomes, making it challenging to draw definitive conclusions regarding the long-term benefits of DHEA supplementation. Safety data on long-term DHEA supplementation are also sparse. Some studies have reported side effects, such as hormonal imbalances, which could potentially increase the risk of adverse events. Therefore, monitoring is essential for individuals undergoing DHEA supplementation over extended periods. A few studies have suggested positive effects on bone health markers; however, they often lack sufficient duration and participant numbers to establish robust long-term efficacy. For instance, while short-term studies indicate improvements in BMD, the absence of comprehensive longitudinal data makes it difficult to confirm sustained benefits or safety. To address these gaps, more extensive and long-term randomised control trials are necessary to evaluate the effectiveness of DHEA supplementation in preventing osteoporosis and fractures, as well as to assess its safety profile in diverse populations.

## 5. Benefits and Risks of DHEA

DHEA is a precursor to oestrogen and androgens, both of which are essential for bone health. It stimulates the production of osteoblasts (bone-forming cells) and influences the expression of bone growth factors through its metabolites. Additionally, DHEA can modulate the activity of osteoclasts (bone-resorbing cells). Research suggests that DHEA can increase BMD and reduce fracture risk by supporting the anabolic (bone-building) effects of oestrogen. The decline in both oestrogen and androgen levels after menopause or with ageing accelerates bone loss, making DHEA supplementation a potential option for restoring hormonal balance. Unlike direct oestrogen therapy, which increases the risk of breast cancer and endometrial hyperplasia, DHEA’s tissue-specific conversion minimises these risks by providing subtler hormonal support without overstimulating oestrogen-sensitive tissues [58]. DHEA supplementation may slow bone loss in women with low DHEA levels, though the optimal dosage and long-term safety remain uncertain. It could complement other treatments, such as bisphosphonates or selective oestrogen receptor modulators (SERMs), to strengthen bones and reduce fracture risk in osteoporosis patients. Combining DHEA with therapies like vitamin D and calcium supplementation may yield synergistic effects on bone health, as vitamin D enhances calcium absorption, which is crucial for bone mineralisation, while DHEA supports the hormonal pathways involved in bone formation [59]. Engaging in weight-bearing exercises alongside DHEA supplementation may further improve or maintain BMD, as physical activity stimulates osteoblast activity, promoting bone growth.

However, it is essential to consider the risks associated with DHEA supplementation. A study by Omura et al. demonstrated that excessive DHEA intake is associated with reduced telomere length in normal cells and increased telomere length in cancer cells, potentially raising the risk of cancer. This suggests that excessive DHEA may stimulate oestrogen-sensitive tissues in certain individuals [60]. Furthermore, a nested case–control study within the European Prospective Investigation into Cancer and Nutrition found that higher concentrations of DHEAS, androstenedione, testosterone, and free testosterone were significantly associated with an increased risk of breast cancer, with relative risks ranging from 1.69 to 2.50 [61]. Other potential risks include liver function disturbances. Long-term DHEA supplementation may affect liver function due to the liver’s role in metabolising hormones. The liver processes DHEA and its metabolites, which could result in liver strain or dysfunction, especially with prolonged or high doses. Potential liver-related side effects include elevated liver enzymes, which could indicate liver damage. This risk is higher in individuals who already have liver conditions or those using other medications that affect liver function. In addition, DHEA supplementation may interact with medications, particularly those for diabetes, by affecting blood sugar regulation. It can reduce insulin sensitivity, leading to higher glucose levels, especially in individuals with pre-existing insulin resistance [62]. Additionally, excess DHEA may disrupt cortisol regulation and the HPA axis, potentially raising cortisol levels. This can exacerbate blood sugar issues and contribute to adverse effects such as increased stress responses, immune suppression, weight gain, and compromised bone health.

Supplementation with DHEA may lead to elevated testosterone levels, particularly in individuals with lower baseline levels. While this can be beneficial in some cases, excessive testosterone can cause side effects such as acne, hair loss, mood swings, and an increased risk of prostate issues in men. Since DHEA can convert to both androgens and oestrogens, individuals may experience fluctuations in hormone levels that could result in conditions such as oestrogen dominance or androgen excess, leading to various health issues. Changes in hormonal levels due to DHEA supplementation may exacerbate mood swings, anxiety, or depressive symptoms. The impact of altered testosterone and oestrogen levels on mood and behaviour is a concern, particularly in vulnerable populations. The lack of long-term studies on the safety of DHEA supplementation raises significant concerns. Without comprehensive data, the potential for unknown long-term consequences on endocrine function remains troubling. In addition, individual responses to DHEA supplementation can vary based on genetic, lifestyle, and health factors. This variability means that some individuals may experience adverse effects while others may not, complicating the assessment of DHEA’s safety profile. Given the broad potential for drug interactions, the decision to use DHEA should be carefully considered, and individuals are advised to consult healthcare providers to weigh the specific risks and benefits related to their health conditions.

## 6. Perspective and Limitation

**Figure 2:**
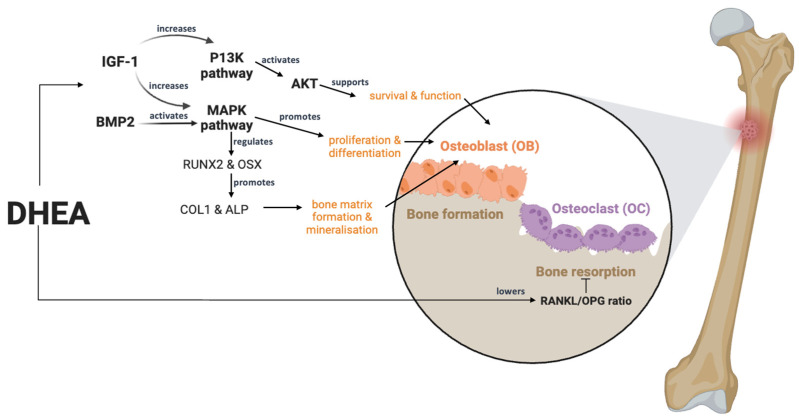
Mechanism effect of DHEA on bone health. ALP, alkaline phosphatase; BMP2, bone morphogenetic protein 2; COL1, collagen type I alpha-1 chain; IGF-1, insulin-like growth factor 1; MAPK, mitogen-activated protein kinase; P13K, phosphoinositide 3-kinase; RANKL, receptor activator of nuclear factor kappa-Β ligand; RUNX2, runt-related transcription factor 2; OPG, osteoprotegerin; OSX, osterix. Created in BioRender. Chua, E. (2024) https://BioRender.com/u84m205 (accessed on 23 October 2024).

The effects of DHEA on bone cells have been previously investigated. DHEA has been shown to stimulate osteoblast differentiation and proliferation, as evidenced by increased expression of osteoblast marker genes (e.g., RUNX2, OSX, COL1) and enhanced ALP activity. The osteogenic effects of DHEA are mediated through various signalling pathways, as shown in Figure 2, including IGF−1, MAPK, PI3K, and BMP2 signalling. DHEA can also modulate the RANKL/OPG ratio, thereby inhibiting osteoclastogenesis and bone resorption. RANKL is a key cytokine primarily produced by osteoblasts and activated T cells. It binds to the RANK receptor on osteoclast precursors, promoting their differentiation into mature osteoclasts, which are responsible for bone resorption. Meanwhile, OPG is a soluble decoy receptor that binds to RANKL, preventing it from activating RANK. This inhibition reduces osteoclastogenesis and bone resorption. DHEA has been shown to downregulate the expression of RANKL. By decreasing RANKL levels, DHEA reduces the signalling that leads to the maturation and activation of osteoclasts. In addition, DHEA appears to enhance the production of OPG. Increased OPG levels inhibit RANKL’s action by binding to it, thus shielding osteoclast precursors from differentiating into bone-resorbing osteoclasts. The exact molecular pathways through which DHEA exerts these effects are still being elucidated. Potential mechanisms may involve DHEA’s interaction with androgen and oestrogen receptors, as it can be converted into these hormones. This conversion likely influences the expression of RANKL and OPG at the transcriptional level. Additionally, DHEA may exert its effects through signalling pathways related to inflammation and osteogenesis, impacting the balance between bone formation and resorption. By inhibiting osteoclastogenesis and promoting a favourable RANKL/OPG balance, DHEA may help maintain or improve bone mineral density and overall bone health in both males and females. This is particularly important for postmenopausal women, who experience increased bone resorption due to decreased oestrogen levels, leading to a higher risk of osteoporosis. While both males and females can benefit from DHEA’s effects, the response may differ due to variations in baseline hormone levels and the presence of oestrogen and testosterone. In males, DHEA may counteract age-related bone loss by modulating the RANKL/OPG axis, while in females, it may offer a therapeutic avenue to mitigate postmenopausal bone loss. DHEA has also demonstrated the ability to counteract the negative effects of glucocorticoids and inflammatory conditions (e.g., *Brucella abortus* infection) on osteoblast function and survival. Interestingly, the osteogenic effects of DHEA appear to extend beyond the mesenchymal stem cell stage, as it can also directly stimulate the proliferation and differentiation of mature osteoblasts.

DHEA activates the Wnt signalling pathway, which stabilises β-catenin in the cytoplasm, leading to its accumulation in the nucleus. This process stimulates the transcription of target genes that promote osteoblast differentiation, including osteogenic markers such as RUNX2 and OSX. Consequently, DHEA enhances mineralisation and bone formation by increasing osteoblast activity and proliferation while inhibiting RANKL expression, which reduces osteoclastogenesis and bone resorption [63,64]. DHEA influences the Notch signalling pathway, which plays a critical role in cell fate determination. Previous studies showed that DHEA upregulated genes linked to bone formation, such as Notch 2 and insulin receptor [22]. By activating Notch, DHEA promotes the expression of genes that encourage osteoblast differentiation while simultaneously inhibiting osteoclast formation. This dual action supports bone formation and helps maintain bone density. In addition, DHEA activates various MAPK pathways, including ERK1/2, p38 MAPK, and JNK, which are essential for cell proliferation, differentiation, and survival [25]. This activation promotes osteoblast differentiation by facilitating the phosphorylation of transcription factors, leading to increased expression of osteogenic markers and enhanced bone formation. Additionally, DHEA’s modulation of MAPK signalling can inhibit osteoclast activity, thereby reducing bone resorption. DHEA also activates the PI3K pathway, which is crucial for cell survival and growth. This activation promotes osteoblast proliferation and survival via downstream effectors such as AKT, resulting in increased expression of osteogenic genes and enhanced matrix mineralisation. Furthermore, DHEA can inhibit osteoclastogenesis by downregulating RANKL signalling through the modulation of the PI3K pathway [65]. DHEA has been shown to enhance BMP signalling, which is vital for bone formation. This enhancement activates the SMAD signalling pathway, leading to increased expression of osteoblast-related genes and promoting bone mineralisation. As a precursor to oestrogens, DHEA binds to ERs in bone cells. Oestrogen signalling via DHEA promotes osteoblast proliferation and activity, enhancing bone formation.

The studies utilising OVX inbred strains of BALB/c and C57BL/6 mice have provided valuable insights into the effects of DHEA on bone health in the context of reduced ovarian function. The administration of 5 mg/kg per day of DHEA, either orally or intragastrically for 90 days, resulted in an increase in trabecular bone volume in the vertebrae and femur of OVX mice. DHEA also promoted the proliferation of osteoblasts and elevated oestradiol production and aromatase activity, contributing to increased oestrogen concentration within the bone. However, the effects of DHEA on bone were more pronounced in the trabecular bone-rich regions, such as the lumbar spine, compared to the cortical bone-dominant regions, like the femoral diaphysis, which did not show significant changes in morphology or strength. This suggests that DHEA may have a more selective impact on specific bone compartments. The active DHEA metabolite, β-AET, showed promise in mitigating the detrimental effects of thermal trauma on bone, preserving bone mineral content, microstructure, and endochondral growth. However, the therapeutic potential of DHEA and its metabolites in the context of trauma-induced bone loss requires further investigation. The studies in orchidectomised male rats demonstrated that DHEA treatment can restore hormonal balance, improve bone mineral density and content, enhance bone remodelling, regulate the OPG/RANKL axis, and preserve normal trabecular bone architecture. These findings suggest that DHEA may have therapeutic potential in the context of male osteoporosis. Some studies have reported that DHEA can suppress bone growth by inhibiting chondrocyte proliferation and differentiation, primarily through oestrogen receptor-mediated pathways. Additionally, DHEA did not significantly affect the viability of bone marrow-derived monocyte/macrophage precursor cells and did not have a significant impact on the inflammatory markers and osteoclast differentiation in *Brucella*-infected osteocytes. These contradictory findings highlight the cell-type-specific effects of DHEA on bone cells and the need for further research to fully elucidate its mechanisms of action and potential therapeutic applications in the context of bone health and disease.

The intricate interplay of hormones in human bone metabolism may not be fully replicated in ovariectomised rats. For example, human bone health is influenced by a broader range of hormones, including parathyroid hormone, calcitonin, and various growth factors. Therefore, future research should consider these complexities and explore the effects of DHEA in the context of multiple hormonal interactions rather than isolating it in simple models. Bone remodelling dynamics in humans are influenced by many factors such as age, gender, and lifestyle, which may not be accurately represented in ovariectomised rat models. This highlights the need for studies that incorporate these variables to better reflect the human condition. Such studies could provide more applicable insights into how DHEA may function in postmenopausal women. The review mentions that many studies using ovariectomised rats are conducted over relatively short timeframes. This limitation can obscure the long-term effects of DHEA on bone metabolism and may not adequately mimic the chronic nature of postmenopausal bone loss in humans. Therefore, longer-duration studies are needed to assess the sustainability of DHEA’s effects over time. Future research should focus on translational approaches that bridge the gap between animal models and human studies. This could involve using advanced technologies such as imaging techniques or biomarker analysis to study bone metabolism in humans, thereby providing more relevant data to assess the efficacy of DHEA in postmenopausal women.

The randomised, placebo-controlled, double-blind studies involving elderly individuals with low androgen levels have provided valuable insights into the effects of DHEA replacement on BMD. These studies found that DHEA supplementation at doses of 75 mg for men and 50 mg for women significantly increased sulphated DHEA and oestradiol levels and improved BMD in the ultra-distal radius in women and the femoral neck in men. This indicates that DHEA replacement can help restore depleted DHEA and oestradiol levels, as well as maintain and potentially improve bone density in the elderly with age-related decline in sex hormone levels. Another randomised, double-blind, controlled trial involving adults aged 60–88 years with low serum DHEAS levels showed that 50 mg of oral DHEA daily for one year significantly improved BMD in the total hip, trochanter, and shaft subregions compared to placebo. Notably, women experienced a significantly greater increase in lumbar spine BMD than men, suggesting gender-specific differences in the skeletal response to DHEA. Long-term studies on DHEA replacement for primary adrenal insufficiency also demonstrated a significant reversal of the progression of femoral neck BMD reduction in those receiving DHEA. Gender-based analyses revealed a differential effect, with a significant enhancement in BMD in the proximal radius of men but not women. DHEA replacement therapy was also found to have a positive impact on bone turnover markers. DHEA treatment increased the serum levels of DHEAS, sex hormones, and IGF-1 while decreasing SHBG. It also reduced markers of bone resorption (CTX) and formation (bone-specific ALP), suggesting a beneficial effect on bone remodelling. Observational studies have also revealed positive associations between serum DHEAS levels and BMD, particularly at the lumbar spine and femoral sites, in both postmenopausal women and men. The balance between cortisol and DHEAS appears to be an important factor in preserving bone health, especially in older adults. While the majority of studies support the beneficial effects of DHEA on bone health, the magnitude and specificity of these effects may vary depending on factors such as age, gender, and underlying health conditions. The lack of significant effects in certain subgroups, such as men in some studies, highlights the need for further research to fully elucidate the complex and potentially nuanced relationships between DHEA, sex hormones, and bone metabolism. Studies also suggest that DHEA may have a differential impact on specific markers of bone turnover in certain disease contexts like SLE.

Testosterone is the primary androgen responsible for promoting bone density in men. As a precursor to testosterone, DHEA may help increase testosterone levels, particularly in older men with low androgen levels. DHEA’s role in enhancing testosterone production influences osteoblast activity and inhibits osteoclast activity. Oestrogen is critical for bone health, especially in postmenopausal women. When oestrogen levels decline, DHEA supplementation may increase oestradiol levels, compensating for the loss of oestrogen and counteracting increased bone resorption. DHEA’s conversion to oestradiol in women enhances ER activation, promoting osteoblast function and inhibiting osteoclastogenesis, thereby favouring bone formation over resorption. In men, DHEA’s conversion to testosterone activates ARs, promoting bone formation and inhibiting resorption. However, the effects may be more pronounced in younger men, as age-related hormonal changes can alter this response. Bone turnover rates differ between genders, with women experiencing more rapid bone loss after menopause due to decreased oestrogen levels. Understanding gender-specific differences in bone metabolism can guide clinical approaches to DHEA supplementation. Postmenopausal women may benefit more from DHEA to mitigate bone loss, while men may require attention to testosterone levels and androgenic support for optimal bone health.

Animal studies have demonstrated the significant potential of DHEA in enhancing bone health. Effective dosages, such as 5 mg/kg/day for 90 days, have been shown to improve trabecular bone volume, while 20 mg/kg for 8 weeks increased serum oestradiol levels. Furthermore, a dose of 250 mg/kg over 6 months restored bone microstructure in osteoporotic models, and 50 mg administered for 12 weeks enhanced bone remodelling in orchidectomised rats. These findings underscore DHEA’s therapeutic promise in addressing bone health issues across various conditions. In human studies, DHEA supplementation has been associated with significant increases in serum DHEAS, testosterone, and oestrogen levels, along with improvements in bone turnover markers and BMD. Doses ranging from 25 to 200 mg have been explored, with most studies showing positive effects on BMD, particularly at lower doses (25–50 mg) over extended periods. The most commonly studied daily doses for bone health are 50 mg, with some studies also investigating 75 mg daily for men. These doses have demonstrated beneficial effects on BMD in elderly individuals and those with low androgen levels. However, while these results are promising, further research is essential to establish the long-term efficacy and safety of DHEA supplementation across diverse populations and age groups.

Personalised dosing strategies are recommended, where the dosage of DHEA is tailored to individual patient profiles. Factors such as baseline DHEA levels, age-related hormonal changes, and specific health conditions should be considered when determining the appropriate dose. This individualised approach could enhance treatment outcomes by optimising the therapeutic effects while minimising potential side effects, like implementing targeted interventions based on comprehensive patient assessments. These assessments could include hormonal profiling, bone density measurements, and evaluations of metabolic health. By understanding each patient’s unique biology and health status, clinicians can design more effective treatment regimens that account for individual variability in response to DHEA. Future studies should also adopt longitudinal designs to monitor the long-term effects of DHEA on bone metabolism across different age and gender groups. This approach would enable researchers to capture changes over time and assess the sustainability of DHEA’s benefits, providing valuable insights into its role in bone health. In addition, there is a need to control for confounding factors such as lifestyle variables (e.g., diet, exercise, and smoking), comorbid conditions, and concurrent medications. By carefully selecting inclusion and exclusion criteria and collecting comprehensive baseline data, researchers can mitigate the impact of these confounding variables on study outcomes.

Overall, the reviewed studies provide valuable insights into the complex and cell-type-specific effects of DHEA on bone health. While the findings generally support the potential of DHEA as a therapeutic agent for osteoporosis, the selective nature of its effects and the potential for adverse outcomes in certain contexts highlight the need for further research.

## 7. Conclusions

DHEA has been shown to stimulate osteoblast differentiation and proliferation, as evidenced by increased expression of osteoblast marker genes and enhanced alkaline phosphatase activity. The osteogenic effects of DHEA are mediated through various signalling pathways, including IGF-I, MAPK, PI3K, and BMP2 signalling. DHEA can also modulate the RANKL/OPG ratio, thereby inhibiting osteoclastogenesis and bone resorption. Furthermore, DHEA can counteract the negative effects of glucocorticoids and inflammatory conditions on osteoblast function and survival. In ovariectomised mouse models, DHEA administration increased trabecular bone volume, promoted osteoblast proliferation, and elevated oestradiol production and aromatase activity, contributing to increased oestrogen concentration within the bone. DHEA treatment has been shown to restore hormonal balance, improve bone mineral density and content, enhance bone remodelling, regulate the OPG/RANKL axis, and preserve normal trabecular bone architecture in orchidectomised male rats. Randomised, placebo-controlled studies in elderly individuals with low androgen levels have found that DHEA supplementation can significantly increase sulphated DHEA and oestradiol levels, as well as improve BMD in the ultra-distal radius in women and the femoral neck in men. The magnitude and specificity of the effects of DHEA on bone health may vary depending on factors such as age, gender, and underlying health conditions, highlighting the need for further research to fully elucidate its mechanisms of action and optimise its clinical applications.

For future studies, it is recommended to conduct long-term studies that could provide insights into the sustained effects of DHEA supplementation on bone health over time, helping to identify any delayed benefits or adverse effects. Genomic analyses to explore how DHEA influences gene expression related to bone metabolism also need to be conducted. By examining changes in the transcriptome and proteome in response to DHEA treatment, researchers can identify specific pathways and molecular targets involved in its mechanisms of action. Investigating genetic polymorphisms associated with DHEA metabolism and response could help identify individuals who may benefit most from supplementation. This approach could lead to more personalised treatment strategies based on genetic profiles. It is recommended to conduct biomechanical studies that assess not only bone density but also bone quality, including parameters such as the microarchitecture and mechanical properties. These studies can provide a more comprehensive understanding of how DHEA affects bone strength and fracture risk. Advanced imaging techniques, such as high-resolution peripheral quantitative computed tomography (HR-pQCT), should be utilised to visualise changes in bone structure and density in response to DHEA supplementation. Large-scale observational studies are also needed to investigate the relationship between endogenous DHEA levels and bone health outcomes across various populations, including postmenopausal women, older men, and individuals with conditions associated with low bone density (e.g., osteoporosis, glucocorticoid-induced bone loss). Specific studies focusing on at-risk populations, such as individuals with a history of fractures or those with chronic conditions affecting hormone levels (e.g., adrenal insufficiency), could provide valuable insights into the potential benefits and risks of DHEA supplementation.

## Figures and Tables

**Table 1:** Effects of DHEA on bone in in vitro studies.

Type of Cells	Treatment (Concentration)	Findings	References
Human bone marrow stromal, HCC1cell line	DHEA 10^−8^, 10^−7^ M	↔RANKL, ↓OPG, ↔RANKL/OPG mRNA ratio, ↓IL-4, ↓IL-6, ↓IFN-γ, ↓MIF, ↔VEGF	[22]
Murine osteoblastic cells from calvariae of neonatal BALB/c	DHEA 10^−5^–10^−10^ M	↑endoplasmic reticulum area per unit cytoplasmic volume, ↑mitochondrial density, ↑S-phase and G2/M phase, ↓G0/G1 phase, ↑ PI, ↓apoptosis, ↑ phosphorylation of ERKs	[23]
Human sarcoma cell line MG-63 and hBM-MSCs	β-AET 0, 0.1, 1, or 10 μM	↑IL-6, ↑IL-8, ↑OPG, ↑cell proliferation	[24]
Human osteoblastic cell lines, hMG63 (hMG63-ER and hMG63-shER)	DHEA 10^−7^ mol/L	↑ endogenous ER β mRNA levels, ↔ variation of ER α mRNA, ↑ osteoblastic proliferation, ↑ OPG, ↑ ERβ, ↑ pERK1/2, ↔ ERα, ↔ RANKL	[25]
Human osteogenic sarcoma SaOS-2 and hBM-MSCs	DHEA 1, 10, or 100 mM	↓viability rate MSCs and SaOS-2 cells, ↑ALP, ↑RUNX2, ↑SMAD1, ↑OP, ↑OC, ↑osteogenic differentiation	[26]
MSC-derived osteoblasts	DHEA 10^−7^ M	↑ALP activity, ↑bone nodules, ↑collagen I, ↑OC, ↑RUNX2, ↑osterix expression, ↑Foxp3+ Tregs, ↔ adipocytes, ↔ PPARγ	[27]
Human marrow stromal cells (KM101) and hBM-MSCs	DHEA 0.01, 0.1, 1, and 10 nM	↑ ALP activity, ↑ IGF-I gene expression, ↑ ALP, ↑ RUNX2, ↑ COL I genes	[28]
hBM-MSCs	DHEA 1, 10, and 100 μM	↑BMP2, ↑RUNX2, ↑ SPARC, ↑ Ca accumulation	[29]
hBM-MSCs	DHEA 0–100 μM	↑ALP activity, ↑ BMP2, ↑ RUNX2, ↑ COL1a1	[30]
Calvarial osteoblasts isolated from female rats	DHEA 1–500 nM	↑osteoblast proliferation, ↑AP activity, ↑osteoblast ER, ↑ALPL, ↑ OSX expression	[31]
BMMs from femurs and tibias of OVX mice	DHEA 10^−6^–10^−9^ M	↓ TRAP-positive cells, ↔cell viability	[32]
Metatarsal bone rudiments isolated from Sprague Dawley rat foetuses	DHEA 30 nM and 100 nM	↓metatarsal longitudinal growth, ↓height of the growth plate hypertrophic zone, ↓ type X collagen mRNA, ↓ thymidine incorporation and collagen X expression, ↑cell death, ↑caspase 3, ↓Bcl-2 to Bax ratio, ↓NF-κB p65-DNA-binding, ↑ ERE-luciferase	[33]
MC3T3-E1 cell line	DHEA 1 × 10^−8^ M	↑osteoblast proliferation, ↓ osteoblast apoptosis, ↑collagen deposition, ↑Ca deposition, ↔ RANKL, ↑OPG, ↓ RANKL/OPG	[34]
MLO-Y4 cell line	DHEA 1 × 10^−8^ M	↔TNF-α, ↔ IL-6, ↔ RANKL, ↔ MMP-2	[35]

Abbreviation: ALP, alkaline phosphatase; Bax, Bcl-2-associated X protein; BcL-2, B cell lymphoma protein 2; BMM, bone marrow-derived monocyte/macrophage precursor cells; BMP2, bone morphogenetic protein 2; Ca, calcium; COL1a1, collagen type I alpha-1 chain; DHEA, dehydroepiandrosterone; ER, oestrogen receptor; ERE-luciferase, oestrogen-responsive elements–luciferase; ERK, extracellular signal-regulated kinase; Foxp3, transcription factor Foxp3; hBM-MSCs, human bone marrow-derived mesenchymal stem cells; IFN-γ, interferon-gamma; IGF-1, insulin-like growth factor-1; IL-4, interleukin-4; IL-6, interleukin-6; IL-8, interleukin-8; MIF, macrophage migration inhibitory factor; MMP-2, matrix metalloproteinases-2; mRNA, messenger ribonucleic acid; MSCs, mesenchymal stem cells; NF-κB, nuclear factor kappa-light-chain-enhancer of activated B cells; OC, osteocalcin; OP, osteopontin; OPG, osteoprotegerin; OSX, osterix; PI, proliferation index; PPARγ, peroxisome proliferator-activated receptor gamma; RANKL, receptor activator of nuclear factor kappa-Β ligand; RUNX2, runt-related transcription factor 2; SMAD1, suppressor of mothers against decapentaplegic1; SPARC, secreted protein acidic and rich in cysteine; TNF-α, tumour necrosis factor-alpha; TRAP, tartrate-resistant acid phosphatase; Treg, regulatory T; VEGF, vascular endothelial growth factor; ↑, increase; ↓, decrease; ↔, no change.

**Table 2:** Effects of DHEA on bone in in vivo studies.

Type of Animal Models (Age)	Treatment/Intervention (Dose, Route, and Duration)	Findings	References
Ovariectomised inbred strains of BALB/c mice (10–12 weeks old)	DHEA (5 mg/kg per day orally, 90 days)	↑ vertebrae BV/TV, ↑ femur BV/TV, ↑ fluorescence intensity, ↑ positive cell percentage	[23]
Thermal trauma-induced bone loss in male BALB/c mice (12–14 weeks old, 25 g)	β-AET (25 and 50 mg/kg, subcutaneously, 3 times/week, 4 weeks)	↑ wet femur, ↑ dry femur, ↑ femur ash, ↔ whole-body BMC, ↑ bone percentage, ↑ Pm/Ar, ↑ longitudinal growth rate, ↓ endocortical erosion	[24]
Ovariectomised C57BL/6 mice (6–8 weeks old)	DHEA (5 mg/kg per day orally, 3 months)	↑ BV, ↑ BMD, ↑ Tb.N, ↓ Tb.Sp	[27]
Ovariectomised inbred strains of BALB/c mice (10–12 weeks old)	DHEA (5 mg/kg per day intragastrically, 90 days)	↑ vertebrae BV/TV, ↑ femur BV/TV, ↑ E2, ↑ aromatase	[36]
Ovariectomised female Sprague Dawley rats (6 weeks old)	DHEA (20 mg/kg b.wt, intraperitoneally, 8 weeks)	↑ DHEA, ↑ E2, ↑ BMD tibial proximal metaphysis, ↔ BMD diaphysis	[37]
Ovariectomised female Sprague Dawley rats (6 weeks old)	DHEA (20 mg/kg b.wt intraperitoneally, 8 weeks)	↑ BMC, ↔ BMD, ↑ femoral weight wet/body weight, ↔ long and short width and length, ↔ breaking force	[38]
Ovariectomised female Sprague Dawley rats (*n* = 23, 6 weeks old)	DHEA (20 mg/kg b.wt, intraperitoneally, once every 3 days 8 weeks)	↑ BMD at proximal metaphysis, ↔ BMD at proximal diaphysis, ↔ Ca accumulation	[39]
Ovariectomised mice female BALB/c (8 weeks old, 20–30 g)	DHEA (5 mg/kg per day intragastrically, 12 weeks)	↑ BMD femur and vertebra, ↑ BV/TV, ↑ Ob. S/BS, ↑ OS/BS, ↑ MS/BS, ↑ BFR, ↓ Oc. S/BS, ↓ Oc. N/BP, ↓ ES/BS, ↓ CD4^+^T, ↓ TNF-α, ↑ E2, ↔ Ca accumulation	[40]
Osteoporotic adult female Sprague Dawley rats induced with prednisolone (130–150 g)	DHEA (250 mg/kg b.wt orally, 3× per week, 60 days)	↑ OPG, ↓ RANKL, ↑ OC, ↑ 1,25-dihydroxyvitamin D3, ↑ PTH, normal trabecular bone and epiphyseal bony structure	[41]
Orchidectomised male Wistar-Kyoto rats (12–14 weeks old)	DHEA (50 mg implanted subcutaneously, 12 weeks)	↑ testosterone, ↑ PTH, ↑ BMD, ↑ BMC, ↑ OPG, ↓ RANK, ↓ DPD, ↔TRAP-5b, ↔ NTX1, ↑ OC, ↑ ALP	[42]

Abbreviation: ALP, alkaline phosphatase; BFR, bone formation rate; BMC, bone mineral content; BMD, bone mineral density; BV/TV, bone volume/total volume; Ca, calcium; DHEA, dehydroepiandrosterone; DPD, deoxypyridinoline; E2, oestradiol; ES/BS, eroded surface/bone surface; MS/BS, mineralisation surface/bone surface; NTX1, N-terminal crosslinking telopeptide of type 1 collagen; OC, osteocalcin; OPG, osteoprotegerin; Ob. S/BS, osteoblast surface/bone surface; Oc. N/BP, osteoclast number/bone perimeter; Oc. S/BS, osteoclast surface/bone surface; OS/BS, osteoid surface/bone surface; PTH, parathyroid hormone; Pm/Ar, perimeter/area ratio; RANKL, receptor activator of nuclear factor kappa-Β ligand; Tb.N, trabecular number; Tb.Sp, trabecular spacing; TNF-α, tumour necrosis factor-alpha; TRAP-5b, tartrate-resistant acid phosphatase 5b; β-AET, androstenediol; ↑, increase; ↓, decrease; ↔, no change.

**Table 3:** The relationship of DHEA on bone in human studies.

Type of Study	Subject Characteristics		Findings	References
Two-year placebo-controlled, randomised, double-blind study	87 men with low levels of DHEA (average age 60 years)	DHEA tablet 75 mg per day, 24 months	↑ BMD at femoral neck, ↑ sulphated DHEA, ↑ oestradiol	[43]
	57 women with low levels of DHEA (average age 60 years)	DHEA tablet 50 mg per day, 24 months	↑ BMD of the ultra-distal radius, ↑ sulphated DHEA, ↑ oestradiol	
Randomised, double-blinded, controlled trial	70 women and 70 men with low level of DHEAS (aged 60–88 years) Oral DHEA 50 mg/d, 1 year		↑ BMD at the total hip, trochanter, and shaft, ↑ BMD at lumbar spine, ↑ BMD at hip regions	[44]
Cross-sectional study	319 postmenopausal women (average age 68 years)		DHEAS positively correlated with BMD at the lumbar spine, significant regression between DHEAS and BMD at lumbar spine and femoral neck	[45]
Double-blind, placebo-controlled trial	106 male and female participants with Addison’s disease (average age 46 years) Oral micronised DHEA 50 mg, 12 months		↑ DHEA, ↑ BMD at femoral neck, ↔ BMD at total hip, ↔ BMD at lumbar spine, ↔ BMD at radius proximal, ↔ BMD at radius mid, ↔ BMD at radius ulna distal, ↔ total BMD	[46]
Prospective, randomised, double-blinded, placebo-controlled 2-year crossover trial	13 females had mild-to-moderate lupus disease (average age 38.7 years) Prasterone 200 mg/day, 22 weeks		↑ RANKL, ↔ OPG, ↔ OC	[47]
Prospective population-based longitudinal study	1003 postmenopausal women (average age 54.7 years)		↑ DHEAS associated with less bone loss at femoral neck and lumbar spine	[48]
Cross-sectional study	19 menopausal women receiving long-term glucocorticoid medication (aged 50–78 years) DHEA 25–50 mg daily, 12 months		↑ DHEAS, ↑ androstenedione, ↑ testosterone, ↑ IGF-1, ↑ OC, ↑ BMD at lumbar spine and femoral neck	[49]
Cross-sectional study	1300 consecutive healthy Korean men (average age 54.1 years)		DHEAS positively associated with BMD values at all skeletal sites	[50]
Cross-sectional study	294 healthy Korean participants (aged 16–85 years)		DHEAS and IGF-1 positively correlated with BMD at various sites	[51]
International multicentre, prospective study	Older men in United States (*n* = 5994), Sweden (*n* = 3014), and Hong Kong (*n* = 2000) (average age 75.5 years)		DHEAS inversely associated with age, DHEAS marginally associated with BMD at femoral neck and lumbar spine	[52]
Cross-sectional data of a prospective multicentre study	77 premenopausal (average age 38.6–42.5), 237 postmenopausal (average age 59.4–59.7), and 481 men (average age 55.3–56.9)		DHEAS positively associated while cort/DHEAS inversely associated with BMD at lumbar spine	[53]
Four double-blinded, randomised controlled trials	295 women and 290 men with low levels of DHEAS (aged 55–85 years) Oral DHEA 50 mg/d, 12 months		↑DHEAS, ↑ testosterone, ↑ oestradiol, ↑ IGF-1, ↑ BMD at lumbar spine, ↑BMD at trochanter, maintained total hip BMD	[54]
Cross-sectional associations	63 perimenopausal women (aged 45–55 years)		DHEAS positively correlated with minimal bone density of maxillary sinus	[55]
Cross-sectional study	478 healthy community-dwelling postmenopausal women (aged 50–90 years)		DHEA positively related to handgrip and gait speed, DHEA positively related with BMD at spine, and total hip	[56]
Randomised, double-blinded, placebo-controlled trial	58 women and 61 men (aged 60–88 years) Oral DHEA 50 mg/d 12 months		↑ DHEAS, ↑ testosterone, ↑ FTI, ↑E1, ↑ E2, ↑ FEI, ↑ IGF-I, ↓ SHBG, ↔ CTX, ↓ BAP, significantly associated with BMD	[57]

Abbreviation: BAP, bone alkaline phosphatase; BMD, bone mineral density; CTX, C-terminal telopeptide; DHEA, dehydroepiandrosterone; DHEAS, dehydroepiandrosterone sulphate; E1, oestrone; E2, oestradiol; FEI, free oestradiol index; FTI, free thyroxine index; IGF-1, insulin-like growth factor 1; OC, osteocalcin; OPG, osteoprotegerin; RANKL, receptor activator of nuclear factor kappa-Β ligand; SHBG, sex hormone binding globulin; ↑, increase; ↓, decrease; ↔, no change.

## Data Availability

No new data were created or analyzed in this study. Data sharing is not applicable to this article.
